# Gout, and Its Relations to Diseases of the Liver and Kidneys

**Published:** 1888-06

**Authors:** 


*Gout, and its Relations to Diseases of the Liver and Kidneys.* By ROBSON ROOSE, M.D. Fifth Edition. London: H. K. LEWIS. 1888.

In September, 1885, we called attention to an earlier edition of this work. We have now the fifth edition. The results of the author's recent experiences have been added, and the book gives a convenient presentation of the various theories held in connection with the subject.

